# Effects of Rumen-Protected Methionine, Choline, and Betaine Supplementation on Ewes’ Pregnancy and Reproductive Outcomes

**DOI:** 10.3390/vetsci12080723

**Published:** 2025-07-31

**Authors:** Basiliki Kotsampasi, Eleni Tsiplakou, Maria-Anastasia Karatzia, Stavroula Oikonomou, Christina Mitsiopoulou, Dimitris Kalogiannis, Eleni Dovolou, Aristotelis Lymperopoulos, Kyriaki Sotirakoglou, Maria Anastasiadou, George Zervas, Stella Chadio

**Affiliations:** 1 Research Institute of Animal Science, General Directorate of Agricultural Research, Hellenic Agricultural Organization—DIMITRA, 58100 Giannitsa, Greece; karatzia@elgo.gr (M.-A.K.); soikonomou@elgo.gr (S.O.); anastasiadou@rias.gr (M.A.); 2 Laboratory of Nutritional Physiology and Feeding, Department of Animal Science, School of Animal Biosciences, Agricultural University of Athens, Iera Odos 75, 11855 Athens, Greece; eltsiplakou@aua.gr (E.T.); chr_mitsiopoulou@aua.gr (C.M.); gzervas@aua.gr (G.Z.); 3 Laboratory of Anatomy and Physiology of Farm Animals, Department of Animal Science, School of Animal Biosciences, Agricultural University of Athens, Iera Odos 75, 11855 Athens, Greece; kad@aua.gr (D.K.); shad@aua.gr (S.C.); 4 Laboratory of Reproduction, Faculty of Animal Science, University of Thessaly, 41223 Larissa, Greece; entovolou@uth.gr; 5 Division of Animal Science, Department of Agriculture, School of Geotechnical Sciences, International Hellenic University (IHU), 57400 Thessaloniki, Greece; alymperopoulos@ihu.gr; 6 Laboratory of Mathematics and Statistics, Department of Natural Resources Management and Agricultural Engineering, Agricultural University of Athens, 75 Iera Odos, 11855 Athens, Greece; sotirakoglou@aua.gr

**Keywords:** methionine, choline, betaine, ewes, gestation, embryonic loss, birth weight, offspring growth

## Abstract

This study evaluated the effects of maternal supplementation with rumen-protected methionine (RPM), alone or combined with rumen-protected choline (RPC) and betaine (RPB), during the periconceptional and prepartum periods on reproductive outcomes and offspring performance in Chios ewes. One hundred synchronized ewes were assigned to three groups—control (no supplementation), M (5.50 g RPM/day), and MCB (3.50 g RPM, 1.60 g RPC, 0.49 g RPB/day)—from day −14 to +14 relative to mating. Blood was collected on days −14, 0, and +14 for ABTS (2,2′-azino-bis(3-ethylbenzthiazoline-6-sulfonic acid), ferric-reducing ability (FRAP), and malondialdehyde (MDA), on days 18 and 21 for progesterone, and on day 26 for pregnancy-associated glycoprotein (PAG) detection. Thirty days before and up to lambing, the ewes were further divided into C-C, C-M, C-MCB, M-M, M-C, MCB-MCB, and MCB-C subgroups. Embryonic loss did not differ between groups. FRAP was higher (*p* < 0.001) in MCB ewes, and ABTS was lower (*p* < 0.05) in M ewes, in the periconceptional period. Offspring in the C-M, C-MCB, M-M, M-C, and MCB-MCB groups had higher birth weights (*p* < 0.01), along with increased MDA levels (*p* < 0.05). The results suggest that maternal methyl donor supplementation during early and/or late gestation enhances antioxidant status, supports embryonic development, and increases birth weight.

## Simple Summary

The periconceptional and prepartum periods are considered to be crucial gestational periods for embryo establishment and fetal development, and maternal nutrition has been considered to be a significant factor for programming the productive and reproductive efficiency of offspring. Given that choline, betaine, and methionine are metabolically closely interrelated, act as methyl donors, and interact through one-carbon metabolism to modulate metabolism, immune responses, and epigenetic events, the objectives of the present study were to examine their simultaneous effects on pregnancy establishment and on offspring birth weight and development when offered to ewes in a rumen-protected form during the periconceptional and late pregnancy periods. The findings indicate that such supplementation enhances maternal antioxidant status, supports embryonic development, and leads to increased birth weight in offspring. These results suggest that strategic supplementation with methyl donors during critical gestational windows can positively influence reproductive outcomes and offspring development.

## 1. Introduction

Maternal nutrition has been implicated as a significant factor for programming productive and reproductive efficiency in animals [1,2]. According to the developmental programming hypothesis, the periconceptional and pre-implantation embryonic development periods are particularly sensitive to changes in maternal nutrition, being critical for both short- and long-term effects [2,3]. Successful pregnancy establishment depends on appropriate communication between the conceptus and the maternal endometrium in the form of maternal recognition signaling, which is crucial for pregnancy maintenance and the subsequent implantation, placentation, and fetal growth [4]. During the time prior to attachment to the endometrium, the embryo is dependent upon uterine secretions—the so-called histotroph, which provides a number of essential nutrients for its development and survival. Amino acids constitute an important component of the histotroph, and apart from their role in protein synthesis they also serve as functional nutrients in regulating a number of key metabolic pathways [5,6]. Studies in both cattle and sheep have revealed that amino acid concentrations increase in uterine fluids during pregnancy and, in particular, methionine, histidine, and lysine are those with the greatest increase during the time of embryo elongation, indicating a critical role in embryonic development and survival [7].

On the other hand, it is now well accepted that maternal nutrition can affect offspring’s future growth and development by inducing epigenetic alterations in the fetal genome, such as DNA methylation, which, in turn, leads to permanent changes in the phenotype of the offspring. Methylation of DNA depends on the availability of methyl donors, such as methionine and compounds of one-carbon metabolic pathways [8]. Methionine, as a precursor of S-adenosylmethionine (SAM), serves as immediate source of methyl groups and, through the one-carbon cycle, influences different processes with effects on cell proliferation and gene expression in the growing organism [9]. Choline, on the other hand, is an essential nutrient with functional relevance in a wide array of biological pathways, including epigenetic modulation of gene expression [10]. Betaine is a choline metabolite [11] and provides three methyl groups, which can be used in transmethylation reactions for the synthesis of numerous substances [12]. Furthermore, betaine, because of its chemical structure, exhibits the properties of the amino acid glycine. As a result of its dual role (methyl donor and amino acid), betaine is involved in protein and energy metabolism [13]. Pregnancy loss during the early stage of gestation has been recognized as one of the major causes of infertility in ruminants [14], and in sheep the majority of embryonic deaths occur before day 18 of gestation [15].

In vitro studies with bovine embryos highlighted the crucial role of methionine for development from the morula to the blastocyst stage [16], whereas cows fed a methionine-supplemented diet from 30 to 126 days of lactation showed lower pregnancy loss from 21 to 61 days after artificial insemination compared to unsupplemented animals [17]. Data from a recent study with Awassi ewes showed that feeding them rumen-protected methionine from 20 days before mating to 20 days after estrus resulted in a higher lambing rate and number of lambs born for each ewe exposed to the ram [18]. Moreover, the effect of some amino acids, including methionine and compounds of one-carbon cycle metabolism, on offspring birth weight and development has been reported [19,20,21,22]. In addition, supplementation with rumen-protected methionine in cows during late pregnancy has been shown to alter the expression of genes associated with gluconeogenesis and insulin signaling in neonatal calves, which would be advantageous for adaptation to the metabolic demands of extrauterine life [23].

Apart from the above-mentioned effects of methionine, a number of studies also support its role in oxidative stress, as increased methionine supply may enhance the flux of the transsulfuration pathway to increase production of the antioxidants glutathione (GSH) and taurine, thus reducing oxidative stress [24,25].

The periparturient period is extremely crucial with respect to maintaining the health and productivity of dairy ruminants, since an increase in oxidative stress (OS), as a response to metabolic adaptations and lipid mobilization due to energy deficit, may result in an increase in reactive oxygen species (ROS), leading to oxidative damage and cytotoxicity [26,27,28,29].

Numerous studies have shown a positive effect of supplementary methionine and/or choline and betaine on the inflammatory and antioxidant status during periods of stress, such as the periparturient and early weaning periods, mainly in cattle [19,20,30,31,32], but also in ewes and goats [33,34,35].

Apart from the well-documented effect of methyl donors in ameliorating oxidative stress during the periparturient period, data has also indicated that some early reproductive processes, such as oocyte maturation to fertilization and embryo development, are particularly sensitive to ROS and require antioxidants for balanced function [36].

Given that choline, betaine, and methionine are metabolically closely interrelated, the objectives of the present study were to examine their simultaneous effects on pregnancy establishment and on offspring birth weight and development when offered to ewes in rumen-protected form during the periconceptional and late pregnancy periods. We also hypothesized that increasing maternal concentrations of methyl donors in pregnancy would enhance oxidative status during these periods.

## 2. Materials and Methods

### 2.1. Animal Management and Nutritional Treatments

For this study, one hundred diary ewes (Ovis aries) of the Chios breed were used. Before the commencement of the study, the ewes were allocated—after equal distribution relative to age, body weight (BW), and lactation number (LN)—to three dietary groups: control (C; n = 40; age = 2.94 ± 0.56 years; BW = 61.09 ± 0.72 Kg; LN = 1.38 ± 0.49), a methionine group (M; n = 30; age = 3.03 ± 0.55 years; BW = 61.22 ± 0.55 Kg; LN = 1.40 ± 0.54), and a methionine, choline, and betaine group (MCB; n = 30; age = 3.01 ± 0.52 years; BW = 61.16 ± 0.53 Kg; LN = 1.40 ± 0.50). The housing and care of the animals conformed to Directive 2010/63/EU of the European Parliament and of the Council of the European Union (2010) [37] on the protection of animals used for scientific purposes.

The ewes were synchronized for estrus, in season (late June), by inserting intravaginal progestagen sponges (Chronogest CR 20 mg, IntervetInternational B.V., Boxmeer, The Netherlands) for 14 days. At the day of the sponges’ removal (day 0), 400 IU of Pregnant Mare Serum Gonadotrophin (PMSG) (Intergonan, Intervet International B.V., Boxmeer, The Netherlands) was injected, followed by ram introduction.

The study was divided into two experimental periods: the first period (periconceptional) lasted from −14 to +14 days relative to day 0, and the second period (periparturient) lasted from 30 days before parturition to lambing.

During the first experimental period (−14 to +14 days relative to day 0), ewes of the control group (C) were offered a basal diet with no supplementation, those of the methionine group (M) were supplemented with rumen-protected methionine (RPM; DL-Methionine), and ewes of the methionine, choline, and betaine group (MCB) were supplemented with rumen-protected methionine (RPM), rumen-protected choline (RPC), and rumen-protected betaine (RPB) (MCB; DL-methionine, choline chloride, and betaine). Each formula was added in concentrated mix in amounts that would provide ewes with 5.50 g of RPM/ewe/day (M) and 3.50 g of RPM plus 1.60 g of RPC and 0.49 g of RPB/ewe/day (MCB) (Table 1). Due to the lack of established amino acid requirements for small ruminants, methionine supplementation levels in sheep diets were extrapolated from recommendations for pregnant dairy cows (NRC, 2001) [38]. Specifically, the NRC (2001) [38] suggests that methionine should constitute 2.4–2.5% of metabolizable protein (MP) in the diets of pregnant dairy cows. Additionally, the ratio of methionine to choline has been recognized as an important factor; based on current knowledge from the NRC (2001) [38], this ratio is recommended to be approximately 2:1 for pregnant dairy cows. Furthermore, as methionine, choline, and betaine all served as methyl donors in this study, their combined inclusion levels were carefully managed to avoid exceeding the total daily amount of methionine.

Feed was offered in portions on a group basis, twice a day (at 0800 and 1600 h), and the animals had free access to water. The diet consisted of alfalfa hay, wheat straw, and the experimental concentrate mixture. From day +15 (relative to day 0) onwards, all three groups were offered the basal diet with no supplementation, until 30 days before the expected parturition, when the second experimental period started. The experimental diet formulations and quantities offered were calculated to meet the ewes’ requirements according to the National Research Council [41] for the maintenance of pregnant ewes carrying two fetuses. The concentrates’ compositions, as well as their nutrient composition, are given in Table 1. Diet selectivity did not occur, and no orts were left from forages or concentrates after each feeding.

Early pregnancy diagnosis was performed by measurement of plasma progesterone levels on days 0, 18, and 21 and plasma pregnancy-associated glycoprotein (PAG) concentration on day 26 (relative to day 0). Pregnancy and twinning were confirmed by ultrasound examination, 66 days after day 0. Only ewes that were found to be pregnant from the first estrus cycle and carrying two embryos (C = 19; M = 16; MCB = 16) were kept in the study and fed the same control ratio up to the second experimental period, which began 30 days before the expected parturition. In addition, blood samples were also collected on days −14, 0, and +14 (relative to day 0) for the detection of total antioxidant capacity (ferric-reducing ability of plasma (FRAP) and 2,2′-azino-bis(3-ethylbenzthiazoline-6-sulfonic acid (ABTS)) and malondialdehyde (MDA) levels.

At the beginning of the second experimental period, ewes of the C group of the first experimental period were further divided into three treatment subgroups: C-C, with no supplementation in either experimental period (n = 7); C-M, supplemented with RPM (n = 6); and C-MCB (n = 6), supplemented with RPM, RPC, and RPB during the second experimental period. Furthermore, in order to evaluate the effects of supplementation during both experimental periods, a subset of group M was kept unsupplemented (M-C group; n = 8), and the other subset was given RPM (M-M group; n = 8) during the second experimental period. Accordingly, a subset of the MCB group was not supplemented (MCB-C group; n = 8) or supplemented with RPM, RPC, and RPB (MCB-MCB group; n = 8) during the second experimental period. Concentrates of the supplemented groups were formulated to provide the same amounts of RPM, RPC, and RPB per ewe per day, as in the first experimental period. The experimental diet formulations and quantities offered were calculated to meet the requirements of ewes carrying twins [41]. The inclusion levels of methionine, choline, and betaine provided in this phase of gestation were calculated as mentioned before in the early period of gestation (periconceptional). The composition and chemical composition of concentrates given during the last month of pregnancy are shown in Table 2. Feed was provided on a group basis in two equal portions at 0800 and 1600 h daily, and the animals had free access to water. The diet during pregnancy consisted of alfalfa hay, wheat straw, and the experimental concentrate mixture, in adjustable quantities to meet the animals’ requirements. After parturition, the ewes were not supplemented anymore. The composition of the postpartum diet was calculated to meet the requirements of a ewe feeding two lambs.

Lambs were delivered naturally. After delivery, they were dried, ear-tagged, vaccinated, and the date of birth and sex were recorded. The lambs suckled their dams until weaning at about 45 ± 0.91 days of age, and their body weight was recorded. The daily growth rate was calculated from birth to weaning. The adjusted 45-day weaning weight was calculated using the formula [(weaning weight − birth weight)/days of age at weaning] × 45 + birth weight [42]. Blood samples for the evaluation of oxidative stress indicators were collected from ewes one day before the expected parturition and at weaning, and from lambs at weaning.

### 2.2. Sample Analyses

#### 2.2.1. Maternal Progesterone Determination

Blood samples were centrifuged for 15 mins at 2800 rpm, and plasma was collected and stored at −40 °C until assayed. The plasma progesterone concentration was measured by radioimmunoassay using a commercial kit (DIA Source PROG-RIA-CT, Louvain-la-Neuve, Belgium). The intra- and inter-coefficient variation was 4.6% and 7.5%, respectively. The detection limit was 0.05 ng/mL. Animals with progesterone levels ≥1 ng/mL at both 18 and 21 days after mating were considered to be pregnant.

#### 2.2.2. Maternal PAG Determination

For the detection of plasma PAG levels, the commercial ELISA kit DG29 (Conception, Reproduction—Animal, Beaumont, QC, Canada) was used, as described by Nanas et al. (2020) [43]. From 26 days of gestation, assay reliability reached 100% for pregnancy in sheep, in good agreement with the results of El Amiri et al. (2014) [44].

#### 2.2.3. Total Antioxidant Capacity and Oxidative Stress Indicator Measurements in Blood

Total antioxidant capacity and oxidative stress biomarkers were measured spectrophotometrically, as described by Tsiplakou et al. (2017) [33]. More specifically, the FRAP assay was used to measure total antioxidant capacity according to the method described by Benzie and Strain (1996) [45]. The ferric tripyridyltriazine (FeIII-TPTZ) complex was reduced to ferrous tripyridyltriazine (FeII-TPTZ) form and developed an intense blue color, with maximum absorption at 593 nm, and the results were expressed as μΜ ascorbic acid/1 g dry matter. The ABTS radical scavenging assay was based on published methods [46,47]. This method is based on the ability of an antioxidant to discolor the ABTS cation radical, which is previously formed through the reaction between ABTS and potassium persulfate. The lipid peroxidation activity in blood plasma was assayed by measuring malondialdehyde (MDA) according to the method of Nielsen et al. (1997) [48], with some modifications. This assay measures MDA, which reacts with thiobarbituric acid (TBA) to form a pink chromogen (TBARS), which is measured at 532 nm.

### 2.3. Statistical Analysis

Pregnancy rate and embryonic loss data were processed using chi-squared analysis. Results concerning the antioxidant status of ewes in the periconceptional period and pre/after parturition were processed as follows: Two linear regression models with repeated measurements were used to investigate the effects of time and treatment on the parameters ABTS, FRAP, and MDA in ewes, using R (version 4.5.0). The first linear regression model with repeated measurements examined the significance of periconceptional treatment (with three levels: C, M, and MCB) at two time points (0 days and 14 days, relative to sponge removal), while accounting for baseline measurements (at −14 days).

yi = m + ai + li + εi

where yi is a vector of ABTS, FRAP, and MDA for the i-th observation; ai is the matrix of the three following fixed effects: treatment (3 levels: C, M, and MCB), time (2 levels: at 0 days and 14 days), and one covariate variable (the baseline measurement at −14 days); li is the ewe, which is fitted as a random effect with l~N (0, σl2); and finally, the εi is the random residual with ε~N (0, σ2).

The second linear regression model with repeated measurements examined the significance of the treatment at two time points (prepartum and weaning), adjusting for baseline measurements (at −14 days).

yi = m + ai + li + εi

where yi is a vector of ABTS, FRAP, and MDA measurements for the i-th observation; ai is the matrix of the three following fixed effects: treatment (7 levels), time (2 levels: at prepartum and weaning), and one covariate variable (the baseline measurement at −14 days); li is the ewe, which is fitted as a random effect with l~N (0, σl2); and finally, the εi is the random residual with ε~N (0, σ2).

Results concerning offspring antioxidant status and body weight, descriptive statistics, and correlation were calculated for all of the following traits: ABTS, FRAP, MDA, NEFA, birth weight, adjusted weaning weight, and growth rate,

The following linear regression model was used in R to investigate whether the mothers’ treatment and the sex of the offspring were affecting the ABTS, FRAP, MDA, NEFA, birth weight, weaning weight, adjusted weaning weight, and growth rate:

yi = m + ai + li + εi

where yi is a vector of ABTS, FRAP, MDA, NEFA, birth weight, adjusted weaning weight, and growth rate for the i-th individual; ai is the matrix of fixed effects: sex (2 levels: male/female) and mothers’ treatment (7 levels); li is the mother of the offspring, which is fitted as a random effect with l~N (0, σl2); and finally, the εi is the random residual with ε~N (0, σ2). For statistical analysis and visualization, R [49] was used. For all regressions, a probability level of *p* < 0.05 was considered statistically significant.

Finally, for the offspring, Pearson correlation coefficients were calculated between ABTS, FRAP, MDA, NEFA, birth weight, adjusted weaning weight, and growth rate, using only complete cases in R.

## 3. Results

### 3.1. Pregnancy Rate and Embryonic Loss

**Figure 1:**
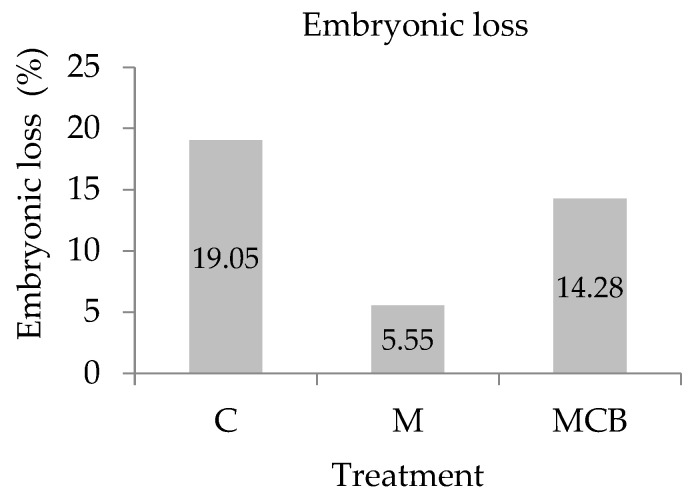
Detection of embryonic loss occurred after estrus synchronization and natural mating.

Ewes that presented with positive progesterone levels but negative PAG levels for pregnancy diagnosis were considered as having undergone early embryonic loss. All animals detected positive by PAGs and confirmed by ultrasound gave birth at the expected date. According to this assumption, early embryonic loss occurred in 5 out of 26 ewes in the control group, in 1 out of 18 ewes in the M group, and in 3 out of 21 ewes in the MCB treatment. Chi-squared test analyses revealed no statistically significant differences (*p* > 0.05) between the three treatments for embryonic loss, which was 5.55% for M, 14.28% for MCB, and 19.23% for C treatment (Figure 1). No significant differences in pregnancy rate were detected among the treatments (C: 65.00%; M: 60.00%, and MCB: 70.00%; *p* > 0.05).

### 3.2. Ewes’ Antioxidant Status Periconceptionally, Prepartum, and at Weaning

Table 3 and Table 4 present the average (average ± SD) values for antioxidant parameters measured periconceptionally, as well as prepartum and at weaning, respectively, while Table 5 and Table 6 present the statistically significant effects of RPM or RPM plus RPC and RPB administration during the periconceptional period, prepartum, and at weaning, respectively.

Summarizing the likelihood ratio tests in ewes for ABTS levels, the baseline measurement of ABTS levels (at −14 days) did not differ significantly in any of the models (periconceptional period, or prepartum and at weaning). During the periconceptional period, a moderate effect (*p* = 0.051; Table 5) of treatment was detected, while time had a significant effect (*p* < 0.01; Table 5). Additionally, a significant interaction between time and treatment was detected (*p* < 0.001; Table 5). Examining the linear regression results for the same period (Table 6), the treatment supplemented with RPM (M) and time (+14 days) had significantly reduced ABTS levels—by −2.65 (*p* < 0.05) and −3.00 (*p* < 0.001), respectively (Table 6)—compared to the control (C) group. At prepartum and weaning, the likelihood ratio tests did not reveal any significant effect of supplementation with methyl donors on ABTS levels (Table 5). According to the linear regression analysis, neither time nor treatment showed a significant effect. However, the observed interaction between MCB (RPM plus RPC and RPB) treatment and time (+14 days) further reduced ABTS levels by −7.08 (*p* < 0.001; Table 6) compared to the control (C).

Regarding FRAP levels, the baseline measurement (at −14 days) did not differ significantly in any of the models (periconceptional period, or prepartum and at weaning). During the periconceptional period, the likelihood ratio tests for the regression indicated that treatment had a significant effect (*p* = 0.027; Table 5) on FRAP levels. Examining the periconceptional regression coefficients (Table 6), treatment supplemented with RPM plus RPC and RPB (MCB) increased (*p* < 0.001) FRAP levels by 0.162 compared to the control (C) group. At prepartum and at weaning, treatment had a significant (*p* < 0.01) effect, while time had a moderate (*p* = 0.08) effect (likelihood ratio tests, Table 5). Additionally, a moderate interaction between time and treatment was observed (*p* = 0.061; Table 5). Furthermore, when examining the regression coefficients, the C-M and C-MCB treatments reduced FRAP levels by −0.187 (*p* < 0.001) and −0.194 (*p* < 0.001), respectively (Table 6), compared to the control group (C). Time also had a reducing effect on FRAP levels by −0.105 (*p* < 0.05) between measurements (Table 6). Statistically significant interaction effects were detected between time and C-MCB, C-M, and M-M (0.253, 0.190, and 0.236, respectively) (Table 6).

Summarizing the results from the likelihood ratio tests for the MDA levels, the baseline measurement of MDA levels (at −14 days) was not significant in any of the models (periconceptional period, or prepartum and at weaning). Periconceptionally, a significant effect of time, but not of treatment, was observed (*p* < 2.2 × 10^-16^, Table 5). Focusing on the results from the regression coefficients (Table 6), time (+14 days) was associated with a reduction in MDA levels (−0.190; *p* < 0.001) compared to the initial measurement. Additionally, prepartum and at weaning, treatment had a significant effect (*p* = 0.01) on their levels, while time had a moderate effect (*p* = 0.057; Table 5). Supplementation with RPM plus RPC and RPB at both experimental periods (MCB-MCB) and RPM alone at both experimental periods (M-M) increased MDA levels compared to the other treatment groups by 0.293 (*p* < 0.01) and 0.341 (*p* < 0.001), respectively (Table 6). Additionally, MDA levels increased by 0.171 at weaning compared to the prepartum measurement (*p* < 0.05). Significant interaction effects were observed between time and the MCB-MCB and M-M treatments (*p* < 0.01 and *p* < 0.05, respectively; Table 6).

### 3.3. Lambs’ Birth and Weaning Body Weights

The average values of offspring’s birth and weaning body weights and growth rates (average ± SD) are presented in Table 7, allowing interpretation of their levels. The subsequent tables (Table 8 and Table 9) present their statistical differences.

The results from the likelihood ratio tests and linear regression for offspring’s birth and weaning body weights and growth rates are illustrated in Table 8 and Table 9, respectively. In all models, the lamb’s sex was statistically significant, except for growth rate. Regarding the mother’s treatment, it had a significant effect on offspring birth weight (*p* < 0.01), but no effect on weaning weight or growth rate. For birth weight, the *p*-value for the mother’s treatment was 0.01 (Table 8). The regression coefficients (Table 9) suggested that the mother’s supplementation in both experimental periods with C-M, C-MCB, M-C, M-M, and MCB-MCB led to a significant increase in offspring birth weight (*p* < 0.001, *p* < 0.001, *p* < 0.01, *p* < 0.01, and *p* < 0.01, respectively).

### 3.4. Lambs’ Antioxidant Status at Weaning

Averages (average ± SD) of lambs’ ABTS, FRAP, and MDA values at weaning are presented in Table 10, followed by Table 11 and Table 12, which illustrate statistical differences.

Table 11 and Table 12 illustrate the results from the likelihood ratio tests, along with each linear regression for ABTS, FRAP, and MDA. In both models, sex had no significant effect (*p* > 0.05). Regarding the mother’s treatment, it had no significant effect on FRAP; however, significant effects on ABTS (*p* < 0.05) and MDA (*p* < 0.01) values were detected (Table 11).

For ABTS, the regression coefficients (Table 12) suggested that the mother’s supplementation with RPM in both experimental periods (M-M) resulted in a statistically significant increase (*p* < 0.01) in offspring ABTS values, compared to all other treatments. For MDA, the regression coefficients (Table 12) revealed that the mother’s supplementation with C-M, C-MCB, M-M, and MCB-MCB led to a significant increase in offspring MDA values (*p* < 0.01, *p* < 0.001, *p* < 0.01, and *p* < 0.01, respectively).

No correlation among the parameters studied (birth weight, adjusted weaning weight, growth rate, ABTS, FRAP, and MDA) was observed according to the results presented in Table 13.

## 4. Discussion

The results of the present study revealed that ewes supplemented with rumen-protected methionine alone during the periconceptional period experienced lower early embryonic loss, as estimated by early pregnancy diagnosis based on progesterone and PAG levels. Although ewes supplemented with a combination of rumen-protected methionine, choline, and betaine also showed reduced embryonic loss, the percentage did not match that observed in the methionine-only group. While this difference did not reach statistical significance, likely due to the small sample size, the nearly fourfold reduction in embryonic losses observed in the methionine-treated group may suggest an improvement in pregnancy establishment. Results with cows have reported that rumen-protected methionine supplementation from 28 until 126 days of lactation resulted in lower pregnancy losses between 28 and 61 days of pregnancy, along with an increase in embryonic abdominal diameter and amniotic vesicle volume on day 33 of pregnancy [17]. Recent data on Awassi ewes reported a higher number of born lambs for ewes supplemented with methionine from 20 days before mating to 20 days after the first estrus [18]. Moreover, rumen-protected methionine and methionine plus choline supplementation from 4 weeks prepartum to 20 weeks postpartum improved the reproductive performance status of dairy cows [50], whereas Raheja et al. (2018) [34] reported significant improvements in the reproductive performance of cows during the hot and humid season when supplemented with betaine. Sows’ dietary betaine supplementation decreased the incidence of early pregnancy failure and increased litter size [51]. Moreover, the addition of a methionine antagonist into the culture medium of bovine embryos has been reported to block the development from the morula to the blastocyst stage [16], indicating that methionine’s metabolism into the one-carbon pathway is crucial for the normal development of embryos. Different levels of methionine supplementation from calving to embryo collection have also been shown to affect the transcriptome of the resultant embryos, inducing important alterations in the expression of genes involved in embryo development and immune response [52]. On the other hand, methionine supplementation at the time of follicular growth and embryo development, even with no effect on methylation, seemed to enhance embryo survival by increasing the lipid contents of blastocysts, which serve as an energy substrate for the pre-implantation embryos [53]. Moreover, supplementation with methionine and choline has been proven to induce a downregulation of pro-inflammatory genes, possibly indicating lower inflammatory processes in follicular cells, as well as higher methionine concentrations in the follicular fluid of methionine-supplemented cows, which can potentially affect oocyte quality [54]. However, the fact that the concurrent supplementation of ewes with rumen-protected methionine, choline, and betaine did not decrease embryonic loss to the same extent as RPM supplementation alone might be due to metabolic interactions among the methyl donors that altered methionine’s availability or utilization during this critical window of early embryonic development. In this regard, the present results may be attributed to metabolic interactions among the methyl donors in the MCB treatment, potentially altering methionine’s utilization or its bioavailability for critical functions such as DNA methylation and antioxidant defense. Previous research has demonstrated that, as choline and methionine participate in the one-carbon metabolism, it is possible that responses to choline are dependent on the supply of methionine, and that animals respond to choline when their diets are not balanced for methionine [55].

The observation of decreased ABTS radical scavenging activity alongside unchanged FRAP values in periconceptionally methionine-supplemented ewes suggests a selective alteration in antioxidant status. On the other hand, the significantly higher FRAP values in ewes supplemented with RPM, RPC, and RPB might indicate that methionine along with choline and betaine can trigger their antioxidant system during the periconceptional period. While both of these findings point to a potential antioxidant mechanism of action, further research is required to determine their impact on reproductive performance under commercial production conditions. Collectively, the data underscore the importance of the periconceptional environment, with early methyl donor supplementation—particularly rumen-protected methionine—appearing to support embryo development, possibly through antioxidant-related pathways. However, given the limited sample size, these findings should be interpreted with caution, and additional studies are warranted to validate the observed reproductive outcomes and clarify the underlying mechanisms.

Accumulating evidence indicates that reactive oxygen species (ROS) and antioxidant systems are important components of reproductive functions, such as ovarian follicular development, ovulation, fertilization, luteal steroidogenesis, endometrium receptivity, and embryonic development [36]. The switch from pre-implantation embryonic anaerobic metabolism to post-implantation aerobic metabolism exposes embryonic cells to ROS produced as byproducts of the enhanced respiratory capacity of embryonic mitochondria [56]. Although the presence of ROS during the first gestational phase is necessary to enhance implantation, a disproportion in ROS generation, if not adequately controlled, could lead to embryo resorption [36]. Therefore, the observed alterations in antioxidant capacity detected in ewes receiving methyl donors during the periconceptional period may have contributed to the control of ROS generation and less embryonic loss. However, due to the small sample size, the results of the present study should be interpreted with caution, and more research is warranted to elucidate the mechanisms involved.

Apart from the periconceptional period, the periparturient period is also crucial, since animals experience metabolic adaptations leading to a negative energy balance and an increase in the production of ROS. Meanwhile, malondialdehyde (MDA), an end-product of ROS-induced lipoperoxidation, can induce oxidative damage and cytotoxicity [57,58], while as pregnancy advances, energetic metabolism undergoes an upregulation, leading to an over-generation of ROS [27]. In our study, the total antioxidant status of ewes, measured by ABTS prepartum and at weaning, was not affected by supplementation. However, ewes supplemented with methionine alone or along with choline and betaine only experienced lower levels of FRAP before parturition, while ewes supplemented with methionine alone or with choline and betaine in both gestational periods experienced higher MDA levels. These findings might show a pronounced oxidative stress because of the longer consumption of additives, or because their already-enhanced oxidative system activated further to support the higher BW of their pregnant fetuses, as indicated from the higher BW at birth. Mavrommatis et al. (2021) [35] found no effect of rumen-protected methionine or/and lysine supplementation, from fifteen days before parturition to sixty days of lactation, on FRAP and ABTS values, but they found lower MDA values in ewes supplemented with methionine alone or in combination with lysine. Similarly, Tsiplakou et al. (2017) [33] found no effect on the mean plasma FRAP and ABTS values in ewes supplemented with protected methionine alone, from fifteen days before parturition to sixty days of lactation. The discrepancies in the results between studies might be attributed to differences in the duration of supplementation, as well as the sampling time.

According to the findings of our study, maternal supplementation with rumen-protected methionine, either alone or in combination with rumen-protected choline and betaine, during the periconceptional, prepartum, or both periods, led to increased birth weights in offspring. In agreement with our results, Liu et al. (2016) [59] reported a 10% increase in the birth weight of lambs born to mothers supplemented with methionine from day 111 of pregnancy until day 7 after lambing. Moreover, Titi et al. (2022) [18] found that maternal rumen-protected methionine supplementation in the periconceptional period and from the last 60 days of pregnancy up to 60 days of lactation increased the birth weight of Awassi lambs. In another study, herbal methionine supplementation of goats during the last third of gestation positively affected the weight and body mass index at birth of the progeny [60]. On the other hand, RPM supplementation of Shami goats in late pregnancy did not affect birth weight of their kids [61]. Supplementation with methionine during the last 30 days of gestation increased calf birth weight [19,21], in good agreement with our results. Jia et al. (2015) [62] also found that the addition of betaine in the diet of sows increased the number of live litters and the litter birth weight, while Gao et al. (2020) [63] found that supplementing maternal diets with betaine hydrochloride significantly reduced piglet mortality and increased the numbers of weaned piglets. Song et al. (2020) [64] reported enhanced development of the heart, kidneys, stomach, and liver of Bama suckling pigs born to mothers fed a betaine diet, which also increased the daily weight gain of piglets. On the other hand, Silva et al. (2021) [65] did not find differences in the birth and weaning weights of calves born to mothers supplemented with rumen-protected methionine during the periconceptional period. These differences may be due to differences between species, or in the dose, time, or duration of supplementation.

The way by which methyl donor supplementation at any stage of gestation leads to increased birth weight is not clear. An enhancement of the uteroplacental intake has been implicated in studies with cattle, in which a greater mRNA expression of genes involved in amino acids and glucose transport, as well as an upregulation of the mammalian target of rapamycin complex (mTOR) pathway in the placenta, was detected after methionine supplementation during the periparturient period [19]. Furthermore, recent data from calves, derived from embryo transfer from choline-treated embryos, reported an increase in birth and weaning weights, underlying the crucial role of the periconceptional environment in programming offspring’s growth and development [66]. In addition, data from pigs supplemented with a cocktail of methyl donors during the periconceptional period reported increased fetal weight at day 90 of age, associated with an increased gene expression of insulin-like growth factor-1 (IGF-1) in fetal muscle tissue [67]. Furthermore, Fu et al. (2022) [51], in their comprehensive review, reported that maternal betaine may regulate glucose and cholesterol metabolism in the fetal liver through epigenetic pathways, thereby affecting the growth and development of the offspring. However, although most of the profound epigenetic changes would be induced during early embryo development, the data from the present study also underline the importance of maternal methyl donor supplementation during early or late pregnancy in eliciting changes in the growth and development of the offspring, as has already been reported for cattle [19,21]. In further support of this hypothesis, methionine administration during the last 30 days of gestation enhanced the maturation of gluconeogenesis and fatty acid oxidation in the livers of neonatal calves, which would be advantageous for adaptation to the metabolic demands of extrauterine life [23].

In the present study, although maternal supplementation with methyl donors enhanced birth weight, only trends for higher growth rate and weaning weight were detected in lambs born to mothers supplemented with methiοnine at any experimental period, or with methionine, choline, and betaine supplemented only periconceptionally. Various studies concerning the effects of maternal supplementation with methyl donors on offspring’s growth potential have shown contradictory results. Suárez et al. (2023) [68] found that supplementation of ewes with biocholine (herbal mix) from mating to 51 days of gestation resulted in a higher birth weight of lambs, but not a higher weaning weight, whereas methionine supplementation of goats in the last trimester of gestation increased the growth variables of the progeny, without any significant influence on the milk yield and composition [60]. Evidence for an increased body weight at birth and 56 days of age has also been reported for calves born to mothers supplemented with methionine for the last 30 days of gestation, suggesting that the mechanisms involved are programmed in utero rather than after birth. Furthermore, various studies have shown that methionine supplementation enhances the immune function and mitochondrial metabolism of offspring [22,69,70], resulting in enhanced postnatal development [21,71]. Moreover, recent results in cattle provide evidence for epigenetic effects, as methionine supply affects the placental metabolism, DNA methylation, and body mass of the calf in a sex-specific manner [72]. High betaine supplementation in ewes (4 g of betaine/ewe/day) in the last month of gestation did not affect lambs’ birth weight or growth rate but resulted in a higher weaning weight [73]. Moreover, ewes’ supplementation with lysine, methionine, and choline from day 80 of gestation to parturition did not alter the lambs’ weights from birth to weaning [74].

In the present study, lambs were nursing from their mothers until weaning; therefore, its effects on postpartum growth and development cannot be disregarded. A lack of an effect on the daily milk yield of goats supplemented with methionine [75] and ewes supplemented with methionine or methionine along with choline and betaine has been reported [33]. Data from a study in cattle sought to examine the effects of colostrum source from methionine-supplemented and -non-supplemented dams on the calves’ postnatal growth response, reporting no significant effects for most of the outcomes measured, indicating little biological effect [21]. More studies are needed to clarify the mechanisms mediating the effects of methionine, choline, and betaine supplementation.

Higher MDA values in offspring born to mothers supplemented with rumen-protected methionine (alone, or with choline and betaine), either during the periconceptional and late gestation periods or only during late gestation, are well correlated with their higher birth weight. Taking this into account, it could be speculated that offspring with higher birth weights likely experienced enhanced nutrient supply and metabolic activity in utero, possibly due to maternal supplementation. This “metabolic programming” can lead to persistent alterations in oxidative metabolism even if postnatal growth is normalized. According to Jacometo et al. (2016) [23], calves born to mothers supplemented with methionine during gestation underwent a faster maturation of gluconeogenesis and fatty acid oxidation in the liver, which would be advantageous for adapting to the metabolic demands of extrauterine life. Therefore, these offspring may retain a higher basal metabolic rate or altered mitochondrial function, leading to more ROS generation, increased lipid peroxidation, and higher MDA levels—even if their body weight gain after birth is similar. To the best of our knowledge, there is no relative data concerning the effects of maternal supplementation with methyl donors during gestation on offspring antioxidant status.

## 5. Conclusions

The results of the present study indicate the effects of maternal supplementation with rumen-protected methionine along with choline and betaine during critical periods of gestation, such as periconception and late gestation, on pregnancy outcomes. Rumen-protected methionine—alone or in combination with choline and betaine, as methyl donors with antioxidant action—seems to play an important role in ensuring embryo survival and improving embryo development, as indicated by the low early embryonic loss and the increased body weight at birth. Thus, the findings of the present study may help in introducing nutritional strategies, such as methyl donor supplementation to improve pregnancy outcomes and embryonic growth.

## Figures and Tables

**Table 1:** Concentrate composition of concentrates offered to periconceptional ewes (−14 to +14 days, relative to estrus synchronization day 0).

	Treatment		
	C	M	MCB
Ingredient Composition (g/kg)			
Corn grain	300	300	300
Barley grain	200	200	200
Wheat grain	200	200	200
Soyameal	100	100	100
Sunflower meal	175	164	157
Mineral and vitamin premix	25	25	25
Methionine formula ^1^	0	11	0
Methionine, choline, and betaine mix ^2^	0	0	18
Chemical Composition (g/kg) ^3^			
Dry matter (DM)	875	864	857
Crude protein (CP)	151	148	145
Crude fat (CF)	19.4	19.2	19.1
Neutral detergent fiber (om)	162	157	154
Acid detergent fiber (om)	81.5	77.9	75.5
Ash	26.8	26.0	25.5
Net energy of lactation (MJ NEL/kg)	6.32	6.26	6.21

Note: ^1^ 550 g of rumen-protected DL-methionine/kg; ^2^ 220 g of rumen-protected DL-methionine, 100 g of rumen-protected chloride choline, and 30 g rumen-protected betaine/kg. ^3^ Concentrates were analyzed for dry matter, crude protein, crude fat, acid detergent fiber, and ash according to the Association of Official Analytical Chemists (1990) [39], and for neutral detergent fiber according to Van Soest et al. (1991) [40]. All other values were calculated from National Research Council (1985) values [41].

**Table 2:** Concentrate composition of concentrates offered to ewes in the last month of gestation.

	Treatment		
	C	M	MCB
Ingredient Composition (g/kg)			
Corn grain	300	300	300
Barley grain	200	200	200
Wheat grain	200	200	200
Soyameal	100	100	100
Sunflower meal	175	165	159
Mineral and vitamin premix	25	25	25
Methionine formula ^1^	0	10	0
Methionine, choline, and betaine mix ^2^	0	0	16
Chemical Composition (g/kg) ^3^			
Dry matter (DM)	875	866	860
Crude protein (CP)	151	148	146
Crude fat (CF)	19.4	19.2	19.1
Neutral detergent fiber (om)	162	158	155
Acid detergent fiber (om)	81.5	78.5	76.7
Ash	26.8	26.1	25.7
Net energy of lactation (MJ NEL/kg)	6.32	6.26	6.23

Note: ^1^ 550 g of rumen-protected DL-methionine/kg; ^2^ 220 g of rumen-protected DL-methionine, 100 g of rumen-protected chloride choline, and 30 g rumen-protected betaine/kg. ^3^ Concentrates were analyzed for dry matter, crude protein, crude fat, acid detergent fiber and ash according to the Association of Official Analytical Chemists (1990) [39], and for neutral detergent fiber according to Van Soest et al. (1991) [40]. All other values were calculated from the National Research Council (1985) values [41].

**Table 3:** Ewes’ periconceptional antioxidant parameters (average ± SD).

Treatment	FRAP −14 (μmol Ascorbic Acid)	FRAP 0 (μmol Ascorbic Acid)	FRAP + 14 (μmol Ascorbic Acid)	ABTS -14 (% Inhibition)	ABTS 0 (% Inhibition)	ABTS + 14 (% Inhibition)	MDA −14 (μM)	MDA 0 (μM)	MDA +14 (μM)
C	0.66 ± 0.18	0.57 ± 0.14	0.55 ± 0.16	41.66 ± 4.34	40.24 ± 3.63	37.53 ± 4.59	0.58 ± 0.08	0.60 ± 0.13	0.57 ± 0.20
M	0.69 ± 0.20	0.62 ± 0.19	0.59 ± 0.24	41.76 ± 3.12	37.67 ± 4.57	35.79 ± 4.83	0.57 ± 0.10	0.54 ± 0.06	0.51 ± 0.09
MCB	0.71 ± 0.20	0.69 ± 0.13	0.79 ± 0.16	40.85 ± 3.74	41.30 ± 2.56	31.95 ± 5.16	0.46 ± 0.04	0.54 ± 0.22	0.42 ± 0.07

**Table 4:** Ewes’ antioxidant parameters prepartum and at weaning (average ± SD).

Treatment	FRAP Prepartum (μmol Ascorbic Acid)	FRAP Weaning (μmol Ascorbic Acid)	ABTS Prepartum (% Inhibition)	ABTS Weaning (% Inhibition)	MDA Prepartum (μM)	MDA Weaning (μM)
C-C	1.08 ± 0.12	0.94 ± 0.21	43.23 ± 5.70	43.98 ± 5.51	0.53 ± 0.14	0.69 ± 0.11
C-MCB	0.88 ± 0.07	0.99 ± 0.16	42.29 ± 6.76	38.17 ± 2.88	0.66 ± 0.18	0.73 ± 0.09
C-M	0.87 ± 0.10	0.96 ± 0.14	43.36 ± 2.83	41.99 ± 2.06	0.62 ± 0.20	0.66 ± 0.08
M-C	1.09 ± 0.18	1.08 ± 0.22	40.92 ± 6.30	47.99 ± 6.41	0.53 ± 0.17	0.66 ± 0.07
M-M	0.97 ± 0.14	1.10 ± 0.15	42.59 ± 6.25	43.40 ± 8.39	0.75 ± 0.27	0.79 ± 0.14
MCB-C	1.05 ± 0.12	0.86 ± 0.11	41.80 ± 8.34	37.34 ± 3.44	0.72 ± 0.22	0.62 ± 0.05
MCB-MCB	0.93 ± 0.07	0.96 ± 0.22	42.95 ± 4.15	39.11 ± 8.84	0.74 ± 0.12	0.70 ± 0.17

**Table 5:** Likelihood ratio tests for the effects of treatment, time, baseline measurement, and their interaction on ABTS, FRAP, and MDA levels in ewes during periconceptional and prepartum–weaning periods.

	ABTS (% Inhibition)		FRAP (μmol Ascorbic Acid)		MDA (μM)	
Periconceptional	Chisq	Pr (>Chisq)	Chisq	Pr (>Chisq)	Chisq	Pr (>Chisq)
(Intercept)	58.31	0.00 *	85.97	<2.0 × 10^−16^ *	15.77	0.00 *
Treatment	5.94	0.051	7.15	0.027 *	4.02	0.13
Time	7.70	0.005 *	0.00	0.98	379,300.00	<2.2 × 10^−16^ *
Baseline measurement	0.55	0.46	0.51	0.48	2.04	0.15
Treatment:time	14.32	0.0007 *	1.12	0.57	1.69	0.43
**Prepartum–Weaning**	**Chisq**	**Pr (>Chisq)**	**Chisq**	**Pr (>Chisq)**	**Chisq**	**Pr (>Chisq)**
(Intercept)	35.59	0.00 *	244.13	<2.0 × 10^−16^ *	2.92	0.09
Treatment	1.22	0.98	16.77	0.01 *	15.99	0.01 *
Time	0.28	0.59	2.90	0.08	3.60	0.057
Baseline measurement	0.00	0.99	0.94	0.33	0.47	0.49
Treatment:time	7.63	0.27	12.02	0.06	10.56	0.10

* Significant estimation.

**Table 6:** Estimated effects and standard errors for treatment, time, and their interactions on ABTS, FRAP, and MDA levels in ewes during the periconceptional and prepartum–weaning periods.

	ABTS (% Inhibition)		FRAP (μmol Ascorbic Acid)		MDA (μM)	
	Effect	SE	Effect	SE	Effect	SE
Periconceptional						
M	−2.647 *	1.39	0.063	0.05	−0.058	0.08
MCB	1.438	−1.57	0.162 ***	0.06	−0.265	0.13
Time (+14)	−3.001 ***	1.08	0.001	0.04	−0.190 ***	0.0003
M:time (+14)	1.125	1.90	−0.023	0.07	0.106	0.10
MCB:time (+14)	−7.085 ***	2.08	0.073	0.08	0.13	0.17
Prepartum–Weaning						
C-M	1.107	2.56	−0.187 ***	0.07	0.076	0.09
C-MCB	−1.145	2.67	−0.194 ***	0.07	−0.052	0.17
M-C	−1.795	2.76	0.048	0.07	0.199	0.13
M-M	−0.518	2.77	−0.095	0.07	0.341 ***	0.10
MCB-C	−0.811	3.10	−0.026	0.08	0.287	0.18
MCB-MCB	−0.73	2.68	−0.114	0.07	0.293 **	0.12
Time (weaning)	1.111	2.08	−0.105 *	0.06	0.171 *	0.09
C-MCB:time (weaning)	−4.857	3.46	0.253 **	0.10	0.259	0.24
C-M:time (weaning)	−1.623	3.32	0.190 *	0.09	−0.133	0.14
MCB-C:time (weaning)	−4.233	3.95	−0.027	0.11	−0.271	0.24
MCB-MCB:time (weaning)	−4.477	3.46	0.109	0.10	−0.398 **	0.16
M-C:time (weaning)	4.171	3.632	0.055	0.107	−0.178	0.17
M-M:time (weaning)	−0.298	3.52	0.236 **	0.104	−0.251 *	0.14
Baseline periconceptional	0.084	0.11	−0.06	0.08	−0.573	0.40
Baseline prepartum–weaning	0.003	0.16	−0.084	0.08	0.241	0.35
Constant periconceptional	36.798 ***	4.819	0.593 ***	0.064	0.940 ***	0.237
Constant prepartum–weaning	42.995 ***	7.207	1.126 ***	0.072	0.377 *	0.22

Note: * *p* < 0.05; ** *p* < 0.01; *** *p* < 0.001.

**Table 7:** Lambs’ birth and (adjusted to 45 days of age) weaning body weights and growth rates (average ± SD kg).

Treatment	Birth Weight (Kg)	Weaning Weight (Kg)	Growth Rate (g/day)
C-C	3.35 ± 0.45	12.31 ± 2.95	0.197 ± 0.061
C-M	4.31 ± 0.29	13.43 ± 3.51	0.201 ± 0.075
C-MCB	4.11 ± 0.63	12.99 ± 2.70	0.198 ± 0.059
M-C	4.08 ± 0.57	13.74 ± 2.81	0.218 ± 0.064
M-M	3.83 ± 0.74	14.16 ± 2.21	0.226 ± 0.048
MCB-C	3.89 ± 0.93	14.69 ± 1.87	0.230 ± 0.037
MCB-MCB	3.87 ± 0.84	12.10 ± 2.47	0.184 ± 0.053

**Table 8:** Likelihood ratio tests for the effects of treatment and sex on offspring birth weight and weaning weight (adjusted to 45 days of age).

Dependent Variable	Birth Weight (Kg)			Weaning Weight (Kg)			Growth Rate (g/day)		
	Chisq	Df	Pr (>Chisq)	Chisq	Df	Pr (>Chisq)	Chisq	Df	Pr (>Chisq)
(Intercept)	357.78	1	<0.000 ***	249.07	1	<2.0 × 10^−16^ ***	141.38	1	<2.0 × 10^−16^ ***
Sex	11.46	1	0.00 ***	4.75	1	0.02915 *	3.18	1	0.07451
Treatment	16.05	6	0.01 **	7.25	6	0.29778	5.58	6	0.471

Note: * *p* < 0.05; ** *p* < 0.01; *** *p* < 0.001.

**Table 9:** Estimated effects and standard errors of treatment and sex on offspring birth weight and weaning weight (adjusted to 45 days of age).

Dependent Variable			Birth Weight (Kg)	Weaning Weight (Kg)	Growth Rate (g/day)
Sex	Male	Coef	0.31 ***	1.3 **	0.023
		SE	0.09	0.50	0.012
Treatment	C-M	Coef	0.91 ***	1.49	0.01
		SE	0.27	1.2	0.025
	C-MCB	Coef	0.88 ***	0.7	−0.0005
		SE	0.27	1.10	0.02
	M-C	Coef	0.61 **	1.61	0.024
		SE	0.28	1.27	0.026
	M-M	Coef	0.60 **	1.83	0.028
		SE	0.26	1.07	0.022
	MCB-C	Coef	0.51	2.69	0.04
		SE	0.33	1.75	0.037
	MCB-MCB	Coef	0.56 **	−0.26	−0.01
		SE	0.27	1.07	0.022
Constant		Coef	3.28 ***	11.81 ***	0.188 ***
		SE	0.17	0.74	0.015

Note: ** *p* < 0.05; *** *p* < 0.001.

**Table 10:** Lambs’ parameters at weaning (average ± SD).

Treatment	ABTS (% Inhibition)	FRAP (μmol Ascorbic Acid)	MDA (μM)
C-C	39.7 ± 6.14	1.09 ± 0.20	0.86 ± 0.19
C-MCB	43.0 ± 8.36	1.24 ± 0.23	1.57 ± 0.40
C-M	43.8 ± 5.91	1.14 ± 0.37	1.22 ± 0.52
M-C	34.8 ± 4.27	1.01 ± 0.15	1.30 ± 0.06
M-M	45.5 ± 5.38	1.16 ± 0.24	1.09 ± 0.09
MCB-C	41.9 ± 0.83	0.82 ± 0.09	1.35 ± 0.11
MCB-MCB	43.7 ± 4.42	1.03 ± 0.12	1.25 ± 0.19

**Table 11:** Likelihood ratio tests for the effects of treatment and sex on antioxidant parameters of the offspring at weaning.

Dependent Variable	ABTS (% Inhibition)			FRAP (μmol Ascorbic Acid)			MDA (μM)		
	Chisq	Df	Pr (>Chisq)	Chisq	Df	Pr (>Chisq)	Chisq	Df	Pr (>Chisq)
(Intercept)	464.65	1	<2.0 × 10^−16^ ***	233.55	1	<0.00 ***	47.73	1	4.89 × 10^−12^ ***
Sex	0.23	1	0.63	3.34	1	0.07	1.47	1	0.23
Treatment	14.51	6	0.02 *	7.06	6	0.32	16.85	6	0.009 **

Note: * *p* < 0.05; ** *p* < 0.01; *** *p* < 0.001.

**Table 12:** Estimated effects and standard errors of treatment and sex on antioxidant parameters of the offspring at weaning.

Dependent Variable			ABTS (% Inhibition)	FRAP (μmol Ascorbic Acid)	MDA (μM)
Sex	Male	Coef	0.78	−0.11	−0.16
		SE	1.61	0.06	0.14
Treatment	C-M	Coef	3.94	0.08	0.41 **
		SE	2.71	0.12	0.19
	C-MCB	Coef	3.33	0.12	0.71 ***
		SE	2.69	0.11	0.18
	M-C	Coef	−5.01	−0.06	0.29
		SE	3.15	0.13	0.24
	M-M	Coef	5.84 **	0.06	0.45 **
		SE	2.60	0.11	0.20
	MCB-C	Coef	2.11	−0.28	0.44
		SE	−4.49	0.18	0.36
	MCB-MCB	Coef	3.91	−0.07	0.50 **
		SE	2.42	0.11	0.25
Constant		Coef	39.36 ***	1.15 ***	0.91 ***
		SE	1.83	0.08	0.13

Note: ** *p* < 0.01; *** *p* < 0.001.

**Table 13:** Correlation among the traits studied in offspring.

	ABTS (% Inhibition)	FRAP (μmol Ascorbic Acid)	MDA (μM)	Birth Weight (Kg)	Weaning Weight (Kg)	Growth Rate (g/day)
ABTS	1.00	0.22	0.13	0.28	−0.09	−0.16
FRAP		1.00	0.16	−0.10	−0.32	−0.30
MDA			1.00	0.18	−0.28	−0.32
Birth weight				1.00	0.12	−0.11
Weaning weight					1.00	0.97
Growth rate						1.00

## Data Availability

The original contributions presented in this study are included in the article. Further inquiries can be directed to the corresponding author.
